# The absolute number of T lymphocyte subsets is beneficial for differential diagnosis of myelodysplastic syndrome with pure red cell aplastic anemia: a case report and review of the literature

**DOI:** 10.3389/fimmu.2025.1552000

**Published:** 2025-04-29

**Authors:** Liangjun Zhang, Huixiu Zhong

**Affiliations:** Department of Laboratory Medicine, Zigong First People’s Hospital, Zigong, Sichuan, China

**Keywords:** T lymphocyte subsets, myelodysplastic syndrome, pure red cell aplasia, differential diagnosis, treatment

## Abstract

The two diseases of myelodysplastic syndrome (MDS) and pure red cell aplasia (PRCA) are independent of each other and can be linked in some cases. Their diagnosis and differential diagnosis are very confusing. Therefore, in order to understand the relationship between MDS and PRCA and improve the diagnosis and treatment of MDS in patients with PRCA, we present a case study of a 71-year-old male patient with anemia. The result of the morphological examination of bone marrow, whole-genome microarray, and bone marrow biopsy all supported the diagnosis of MDS at the first clinical diagnosis. Azacitidine and venetoclax chemotherapy were given to the patient. However, the treatment is not effective, and the absolute number of T lymphocyte subsets decreased gradually during treatment. Then, the treatment plan was changed to cyclosporine A plus prednisone for immune regulation. The absolute number of T lymphocyte subsets and hemoglobin (Hb) rose rapidly, and the final diagnosis of the patient was MDS with PRCA. To improve the ability to diagnose MDS with PRCA, we should combine it with the absolute number of T lymphocytes to monitor efficacy evaluation during treatment, which contributes to the differential diagnosis of MDS with PRCA.

## 1 Case

Myelodysplastic syndrome (MDS) is characterized by cytopenia and dysplasia in one or more of the major myeloid lineages, ineffective hematopoiesis, and recurrent genetic abnormalities; the main clinical features are pathological hematopoietic in bone marrow, decreased three lineage cells in peripheral blood, and high risk of transformation to acute myeloid leukemia (AML). Pure red cell aplasia (PRCA) shows the hematopoietic failure of the erythroid lineage caused by multiple causes. The main symptom is anemia with lower-than-normal hemoglobin (Hb) in peripheral blood; the reticulocyte decreases, but the leukocytes and platelets are normal; erythroblast is significantly reduced or absent in bone marrow; three lines of cells are without pathological hematopoietic, rare cytogenetic abnormalities (1). MDS is easily misdiagnosed as PRCA when it is characterized by red hyperplasia inhibition and no obvious pathological hematopoiesis, while PRCA accompanied by pathological changes is easily misdiagnosed as MDS. However, such cases are relatively rare, while MDS combined with PRCA is even rarer. Therefore, we collected a case of MDS combined with PRCA and described the process of the disease in detail.

**Figure 1:**
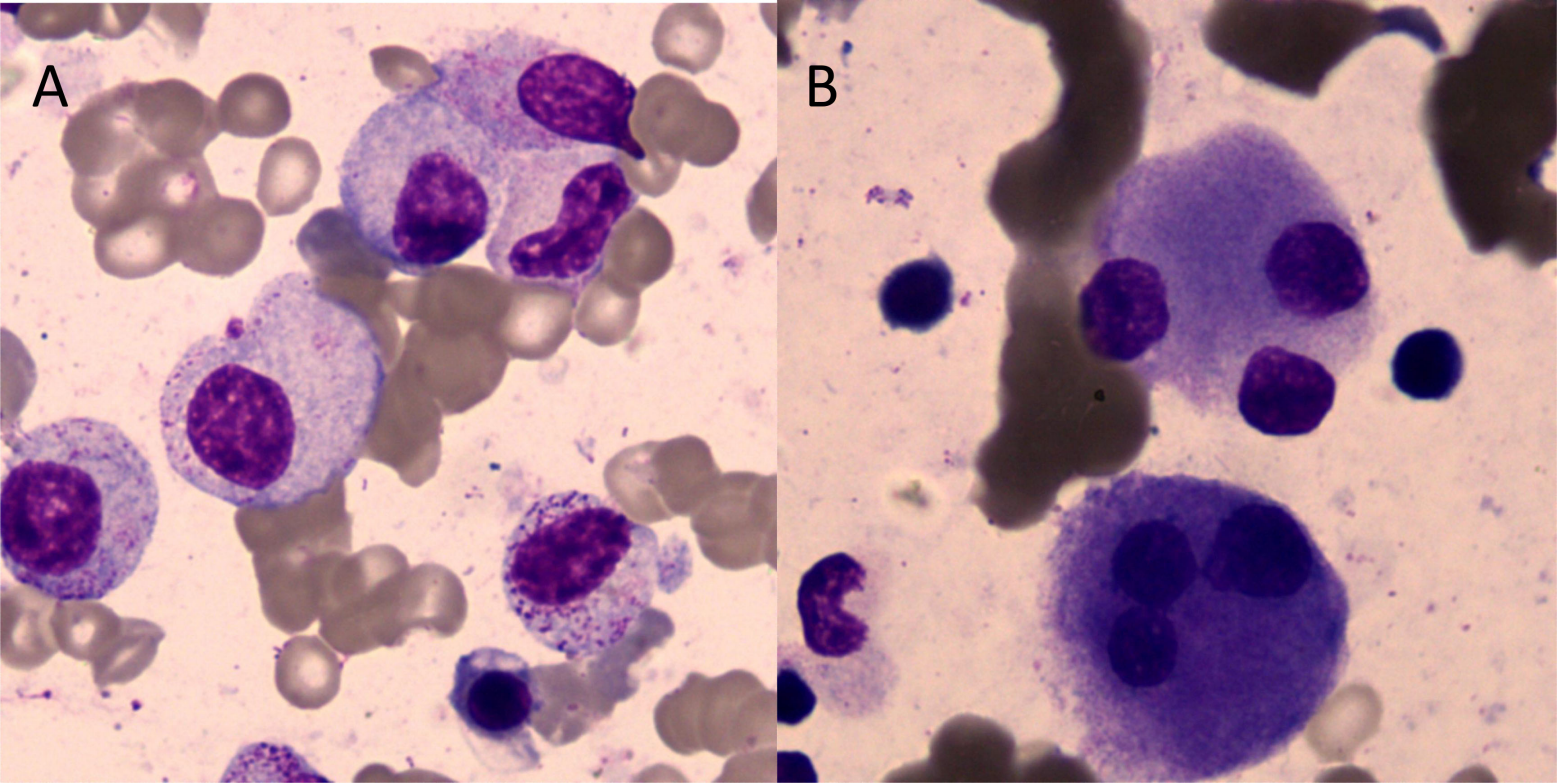
Cell morphology in bone marrow (Wright–Giemsa staining, ×1,000). **(A)** Granulocytes showed that the particles in the cytoplasm were reduced or absent. **(B)** Megakaryocytes showed multi-round megakaryocytes.

**Figure 2:**
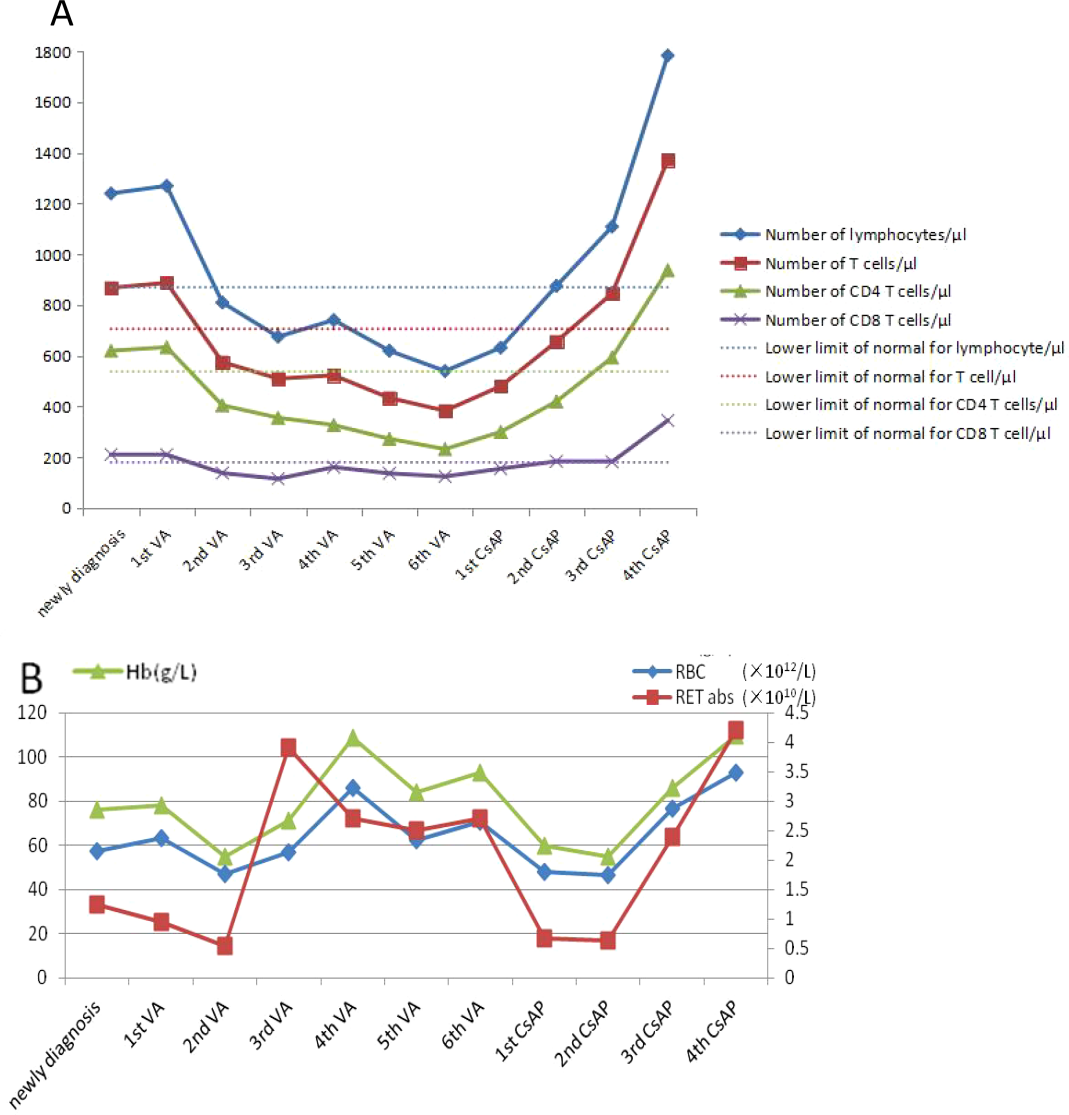
Chart of changes in the index for patients during treatment. **(A)** The test of T lymphocyte subsets. **(B)** The test of blood routine.

A 71-year-old man was hospitalized due to heart fatigue for 0.5 years in September 2022. The blood routine test showed that white blood cells (WBC) of 4.62 × 10^9^/L, red blood cells (RBC) of 1.83 × 10^12^/L, Hb of 67 g/L, platelets (PLT) of 208 × 10^9^/L, and reticulocyte (RET) of 0.04%. Ferritin was 895 ng/mL, and the other tests were all normal. The morphologic test in the bone marrow showed that the percentage of the erythroid cells accounted for only 1.0%; the pathological changes in granulocytes showed that they were small or had significantly large cells, nuclear hyposegmentation with pseudo-Pelger-Huet nuclei, nuclear hypersegmentation, hypogranularity or agranularity of the cytoplasm were observed; the megakaryocytes were easily found because they were micromegakaryocytes and mononucleated and multinucleated megakaryocytes; all their percentages were more than 10% (**Figure 1**). Bone marrow biopsy showed that the proportion of erythroblasts decreased, and megakaryocyte was easily seen as small megakaryocytes, and mononuclear and multinucleated megakaryocytes (pathological megakaryocytes >10%). There were no special abnormalities in immunohistochemical staining, so the diagnostic conclusion of bone marrow biopsy was consistent with MDS. The results of MDS whole-genome chip detection showed the deletion of q35q36.1 on the long arm of chromosome 7. Chromosome karyotype analysis was normal. The patient underwent anemia-related examinations, autoimmune-related examinations, infection-related examinations, heart-related examinations, chest and abdominal CT, electrocardiogram, and heart color ultrasound, and no abnormalities were found in the results of the above tests. The myeloid, lymphoid, and erythroid lineages in bone marrow samples were detected by flow cytometry, and the phenotype and clonality of B/T lymphocytes were normal. The patient had no recent history of medication, so cytopenias caused by other reasons were ruled out; the patient was diagnosed with myelodysplastic syndrome based on clinical and examination results. From October 2022 to June 2023, the patient received six courses of venetoclax + azacitidine (VA) chemotherapy. During chemotherapy, Hb first dropped to 55 g/L, then rose to 109 g/L, and soon dropped. The change trend of RBC and RET absolute value was basically consistent with Hb (**Figure 2**). In T lymphocyte subsets, the absolute number of T lymphoid subsets (including T cells, CD8^+^ T cells, CD4^+^ T cells showed a gradually decreasing trend (**Figure 2**).

After six courses of chemotherapy, the re-examination of the bone marrow showed that the hyperplasia of the erythrocyte system was suppressed (1.5%). Combined with the bone marrow examination results at the initial diagnosis, the possibility of MDS with PRCA could not be ruled out. The dosage was changed to cyclosporine A + prednisone (CsAP) as immune regulation, the patient’s Hb gradually rose to 110 g/L, the absolute number of RET and RBC also increased, and the absolute value of T lymphocyte subsets increased significantly, rising to more than the value at the first diagnosis (**Figure 2**).

## 2 Discussion

MDS can be manifested as one or more lines of hematopoietic dysfunction, the clinical manifestations are varied, and the diagnosis is complicated. Some studies have shown that some MDS patients respond to immunosuppressive or immunomodulatory therapy, which suggests that there is a relationship between MDS and immune abnormalities (2). In recent years, the pathological mechanism of MDS lies in the abnormal activation of inflammation and innate immunity. It has been proposed that in pathological states such as autoimmune abnormalities, chronic infection, and aging, the inflammatory changes in the bone marrow microenvironment may be caused by promoting the abnormal secretion of inflammatory factors and chronic inflammatory stimulation. They may induce abnormal activation of innate immune signals in hematopoietic stem cells of bone marrow, promote ineffective hematopoiesis, and increase the risk of cell gene mutation, all of which lead to the development of MDS (3), but this mechanism has not been confirmed by clinical studies.

Studies have reported that approximately 10% of MDS patients can be secondary to autoimmune diseases, such as rheumatoid arthritis, Bechet syndrome, systemic lupus erythematosus, Sjogren’s syndrome, and vasculitis, and rheumatoid arthritis is more common (4). However, PRCA is extremely rare; it occurs in approximately 2%–6% of patients with MDS (5). Studies have shown that the probability of secondary autoimmune diseases in some MDS patients is higher than its natural incidence, and some patients may have abnormal immunological indicators, although they have no clinical manifestations of immune diseases. This provides further evidence for the link between MDS and autoimmune diseases (6). The pathogenesis of MDS is related to the autoimmunity against hematopoietic stem cells. Some specific immune stimuli may trigger an autoimmune response *in vivo*, resulting in the abnormal clonal development of hematopoietic stem progenitor cells, such as abnormal T-cell response to antigen or abnormal T–B-cell interaction, which can lead to the destruction of hematopoietic stem cells (7, 8). It is also believed that MDS itself can lead to a series of autoimmune abnormalities. MDS cloning abnormalities occur not only in hematopoietic stem cells but also in T and B lymphocytes, such as the reduction of natural killer cells, the activation of B lymphocytes, and damage to macrophages that lead to the obstruction of immune complex clearance, thus leading to immune diseases (9). However, the pathogenesis of PRCA in MDS is unclear. Azacytidine and venetoclax are commonly used first-line drugs in the treatment of MDS. Azacytidine is a cytosine nucleoside analog, and venetoclax is a B-cell lymphoma factor-2 inhibitor, both of which have anti-tumor effects. In this study, after three courses of VA treatment, the hemoglobin of the patient recovered to 109 g/L, indicating that the treatment was effective and that clonal tumor cells may exist in the body, which was consistent with the diagnosis of MDS. However, after six courses of treatment, hemoglobin dropped to 55 g/L again, indicating that immune disorders may play a dominant role in the body after the suppression of clonal tumor cells. Therefore, the effect of VA treatment was not good, and the patient quickly returned to normal, changing to immunomodulator therapy, which proved that the patient indeed had both MDS and PRCA disease at the same time, rather than MDS or PRCA disease alone. This study confirmed that MDS with PRCA is a complex disease, and the treatment method is complicated and changeable. Some studies have also demonstrated this view. Wang, H., et al. found that immunoregulation was effective in some patients with MDS with PRCA, while some patients were not (10). Therefore, it is important to accurately diagnose MDS with PRCA. At present, it is very difficult to distinguish the diagnosis of MDS with PRCA from that of MDS in which there was PRCA as the primary manifestation in the clinic, which was very confusing. A very small number of PRCA patients could have malignant cloning transformation during the disease, which can be transformed into MDS or AML. PRCA disease does not have pathological hematopoiesis; when patients have pathological changes in bone marrow combined with chromosome, gene, and other tests, it is not difficult to diagnose MDS. However, the MDS of our study was PRCA as the first manifestation; was it secondary to PRCA, or was MDS combined with PRCA? Secondary diseases are diseases directly caused by the primary disease or specific factors, and the primary disease starts earlier than the secondary disease. Combined diseases are two or more independent diseases at the same time; they can occur at the same time or successively, and they may affect each other, but there may be no direct causal and pathological relationship between each other (11). It was difficult to distinguish based on laboratory test results alone, and it was complicated and took a long time to diagnose, which was made in combination with the patient’s medical history and outcome of treatment. In this case, we found that after VA chemotherapy, although Hb, RBC, and RET gradually increased, the absolute number of T subgroup cells (including T cells, CD4^+^ T cells, and CD8^+^ T cells) decreased gradually and was much lower than the lower limit of normal reference value, and the disease did not improve; but after CsAP treatment, the absolute number of T subgroup cells rapidly increased, and the disease quickly improved. Some studies have shown that after azacitidine treatment for MDS patients, CD4^+^ and CD8^+^ T cells increased; it not only can restore the hematopoietic function but also reverse the immune derangement typical of these hematologic disorders (12). However, that is not what happened in this case. Therefore, we can monitor the absolute value of the T lymphocyte subsets to determine whether the treatment is effective and whether it is consistent with the diagnosis. This is a very effective method, but it needs to collect a large number of cases to verify for MDS with PRCA.

In conclusion, when erythroblast hyperplasia of bone marrow was suppressed and accompanied by pathological hematopoiesis, the possibility of PRCA should be considered, not only MDS, especially if the efficacy of MDS-related treatment alone was not good. We should pay special attention to whether MDS was combined with PRCA. We should not only use comprehensive analysis from cell morphology, immunology, cytogenetics, and molecular biology (MICM) but also use the absolute number of T lymphocytes to monitor the efficacy evaluation of patients and verify whether the diagnosis is correct. It can improve the diagnosis and treatment level of MDS with PRCA.

## Data Availability

The original contributions presented in the study are included in the article/supplementary material. Further inquiries can be directed to the corresponding author.
